# A Review of Plant-Based Therapies for the Treatment of Urinary Tract Infections in Traditional Southern African Medicine

**DOI:** 10.1155/2021/7341124

**Published:** 2021-07-29

**Authors:** Ian Cock, Nothando Mavuso, Sandy Van Vuuren

**Affiliations:** ^1^School of Environment and Science, Griffith University, Brisbane 4111, Australia; ^2^Environmental Futures Research Institute, Griffith University, Brisbane, Australia; ^3^Department of Pharmacy and Pharmacology, Faculty of Health Sciences, University of the Witwatersrand, Johannesburg, Gauteng 2193, South Africa

## Abstract

Urinary tract infections (UTIs) are amongst the most common bacterial infections globally, with ∼11% of the world's population contracting at least one infection annually. Several South African plants are used in traditional healing systems to treat UTIs, yet the therapeutic potential of these plants against bacteria that cause UTI remains poorly explored. This study documents southern African plant species used traditionally to treat UTIs. An extensive literature review was undertaken to document the southern African plant species that are used in traditional South African medicine to treat UTIs, thereby highlighting gaps in the current research that require further study. One hundred and fifty-three southern African plant species that are used to treat UTIs were identified. Eighty-five southern African plants were identified as having noteworthy inhibitory activity against the major UTI-causing bacteria. Few of those studies screened against all of the bacterial causes of UTIs, and none of those studies examined the mechanism of action of the plant preparations. Furthermore, many of those studies did not test the toxicity of the plant extracts, so an evaluation of the safety for therapeutic usage was lacking. Substantial further research is to determine their potential for therapeutic use.

## 1. Introduction

Urinary tract infections (UTIs) are amongst the most common human infections globally. Indeed, it has been estimated that nearly 800 million people (equating to approximately 11% of the global population) develop at least one UTI in any given year [1, 2]. They are substantially more common in women than in men, with the prevalence in women estimated to be approximately five times higher than in males [3]. Indeed, it is expected that more than half of female population of the world will contract at least one UTI in their lifetime, with a substantial proportion experiencing recurrent infections [1]. With the exception of a spike in UTI occurrence in women aged 14–24 years old, the prevalence of UTIs generally increases with age, with the highest incidence in women over 65 years of age [4]. The difference in rates of UTIs between men and women is related to anatomical differences between the genders. As the urethra is located closer to the anus and is shorter in women than in men, women are substantially more susceptible to infections by uropathogens [5]. Additionally, individual health status affects the incidence of UTIs. For example, immunocompromised individuals and sufferers of chronic uncontrolled diabetes mellitus have substantially increased incidences of UTIs as their weakened immune systems are unable to effectively combat infections [3].

Lifestyle and environmental factors also contribute to the prevalence of UTIs. Older adults often accumulate multiple medical conditions, and their treatment and management regimens may increase the risks of contracting UTIs. In particular, catheterisation substantially increases the incidence of UTIs, especially by Gram-negative bacterial pathogens [1]. Indeed, healthcare-associated UTIs have been estimated to account for approximately 10% of UTI cases, with 75% of these being reported in female patients [6, 7]. Additionally, prolonged antibiotic usage to treat other medical conditions weakens the immune response, thereby increasing the susceptibility to UTIs. In younger women, increased sexual activity between the ages of 18 to 39 years of age increases both the incidence of UTIs and the frequency of recurrence [4]. Any region of the urinary tract may become infected, including the kidneys, bladder, urethra, and ureter [8]. When the UTIs occur in the lower regions of the urinary tract, the infection is known as a bladder infection (cystitis). Infections in the upper urinary tract (pyelonephritis) are commonly referred to as kidney infections.

### 1.1. Types of Urinary Tract Infections

Urinary tract infections are classified as either complicated or uncomplicated. Complicated infections occur in people with underlying conditions or abnormalities in any part of the genitourinary tract, making the infection more serious and more challenging to treat than uncomplicated infections. In contrast, uncomplicated UTIs are classified as infections occurring in the absence of comorbidities or other anatomical urinary tract and renal abnormalities [9]. The incidence of complicated UTIs is substantially lower than that of uncomplicated UTIs, which occur in otherwise healthy people with normal genitourinary tract anatomy [10]. However, uncomplicated infections are generally easier to manage, and treatment with a short course of antibiotics is usually effective. Urinary tract infections in children and males are generally categorised as uncomplicated infections due to their low probability of comorbidities [8]. Notably, complicated UTI-causative pathogens are linked to increased rates of antimicrobial resistance. Therefore, the development of effective therapies to treat these conditions is vital, not only to decrease the effects of these infections, but also to slow the development of further antibiotic-resistant bacterial strains.

### 1.2. Causes of Urinary Tract Infections

Interestingly, there can be notable differences between the infectious agents responsible for uncomplicated and complicated UTIs. The vast majority of these pathogens are normal components of the gastrointestinal or vaginal microflora, thereby increasing the chances that they cause UTIs. For both classes of UTI, uropathogenic *Escherichia coli* are the leading infective agent, accounting for approximately 75 and 65% for uncomplicated and complicated UTIs, respectively [1]. *Klebsiella pneumoniae* accounts for a further 6% and 8% of uncomplicated and complicated UTIs. The bacterium *Staphylococcus saprophyticus* causes about 6% of uncomplicated UTIs yet does not significantly contribute to complicated UTIs. In contrast, *Enterococcus* spp. contribute substantially to complicated UTI cases (∼11%), yet contribute less to uncomplicated UTIs (∼6%). Other bacteria also contribute significantly, albeit with substantially lower rate, to the incidence of UTIs. In particular, *Proteus* spp. (particularly *Proteus mirabilis*) and *Pseudomonas aeruginosa* each cause approximately 2% of both uncomplicated and complicated cases of UTIs. Other pathogens may occasionally also cause UTIs. For example, *Staphylococcus aureus* induces a low number of cases of UTIs, although these are generally considered a special case as they are usually secondary to blood *S. aureus* infections. UTIs can also be caused by fungal and viral pathogens, albeit with a substantially lower prevalence than reported for bacterial UTIs. In this review, we focus on the major bacterial causes of UTIs. Therefore, whilst numerous studies have screened traditional medicines for the ability to inhibit *S. aureus*, those studies generally focussed on other diseases (e.g., skin disorders), and we have not listed those studies herein due to the minor role of this bacterium in inducing UTIs. Likewise, *Candida albicans* infections are a common cause of urethra infections and are thus commonly classed as urethritis rather than a UTI. Therefore, we do not include studies examining the effects of southern African plants to inhibit *C. albicans* growth in this review. Numerous other studies have examined the effects of southern African plants against *C. albicans*, and the reader is directed to those studies [11–13]. Notably, those studies generally screened against *C. albicans* for reasons not associated with UTIs.

The pathogens that cause UTIs usually enter the urinary tract via the urethra. Bacteria are transferred to the urethra from the bowel. When they colonise the bladder, they attach to the bladder wall and form a biofilm, which helps the pathogens to evade the host's immune response [14]. Improper urogenital area hygiene, sexual intercourse, and exposure to unfavourable hygiene products (e.g., scented and chemical filled feminine products and contraceptives) may aid in the introduction of pathogens to the urinary tract and create suitable growth conditions for infections to develop [10]. Other risk factors for contracting an uncomplicated UTI include sexual intercourse with a new sexual partner, use of contraceptives, and a history of previous recurrent UTIs. Risk factors for complicated UTIs are underlying diseases, use of catheters, abnormal genitourinary anatomy and physiology, hospitalisation, and exposure to antibiotics [10].

### 1.3. Symptoms of Urinary Tract Infections

Urinary tract infections may present in several ways including increased and persistent urgency to urinate, painful burning sensations associated with urination, increased frequency of urination, lower volumes for each urinary event, and cloudy and foul smelling urine. Pain in the lower abdomen, back, and pelvic area is also a relatively common symptom of UTIs, especially in women [8]. Occasionally, UTIs may result in blood in the urine, which may present as red-, pink-, or cola-coloured urine. Infection in the kidney may present with symptoms including nausea and vomiting, fever, and upper back pain (most commonly on a single side) [10]. Many of these signs and symptoms are generic, and UTIs are frequently overlooked or misdiagnosed as other conditions, particularly in older people.

### 1.4. Current Treatments

In most cases, UTIs are relatively easy to treat with a course of broad-spectrum antibiotics, although fluoroquinolones (including ciprofloxacin) are generally avoided as the side effects are often regarded as outweighing the benefits. The most common treatments include trimethoprim/sulfamethoxazole, fosfomycin, nitrofurantoin, cephalexin, and ceftriaxone. However, due to overuse and misuse of commonly used clinical antibiotics, the emergence of antibiotic-resistant pathogens is increasingly common, resulting in the failure of the main antibiotic chemotherapy options [15]. Antibiotic resistance has been a driving force of new drug development initiatives, and implementation of alternative treatment identification and new antibiotic therapies are urgently required.

The development of antibiotic-resistant bacteria is increasingly resulting in antibiotic therapy failures, and chronic UTIs are becoming more frequent [1]. Additionally, the relatively high rate of UTI recurrence poses a challenge to the effective treatment of these infections [16]. Indeed, that study estimated that approximately 24% of people contracting a UTI will develop a recurrent infection within six months of the original infection. Of further concern, approximately 5% of people who develop a UTI will experience more than three recurrences per year [17].

Foxman and Buxton [18] suggest that empirical treatments of UTIs should be reconsidered due to the following reasons:

- The frequency of antibiotic-resistant *E. coli* strains, many of which have resistance to multiple antibiotics including fluoroquinolones [15], is increasing. The increasing incidence of extended spectrum *β*-lactamase (ESBL) UTI pathogens that are resistant to the commonly used *β*-lactam class of antibiotics is particularly concerning [19, 20].
- Even relatively short courses of currently used antibiotic therapies may significantly affect the gut microflora, resulting in other health issues developing [21]. These therapies may also disrupt the urogenital microbiome, resulting in other unforeseen issues. Thus, the benefits of empirical antibiotic therapy may not outweigh the risks [22, 23]. New and innovative strategies to prevent UTI recurrences and alternative therapies for their treatment are considered a high priority [18].

### 1.5. The Use of Medicinal Plants in Urinary Tract Infection Treatment

The launch and development of the WHO Traditional Medicine Strategy 2014–2023 aimed to support the development and implementation of proactive policies and action plans to improve the role traditional medicine plays in population health [24]. The strategy focuses on developing new health systems (including the use of complementary and traditional medicinal products) as a high priority. A re-examination of traditional medicines is an attractive option for the development of new therapies to treat pathogenic infections as plant-derived medicines have often been used for hundreds (in some cases, thousands) of years. Furthermore, the traditional use by some cultures has been relatively well documented. Asian, Middle Eastern, and African traditional systems are perhaps the most extensively documented, although many of the therapies are yet to be extensively studied and verified, and substantially more work is needed in this field.

It is estimated that approximately 700,000 tons of plant materials are used each year in South Africa to produce herbal remedies worth 1.2 to 2.5 billion South African Rands annually [25–28]. Not only are these products widely used in South Africa by practitioners of traditional medicine, but they are also becoming increasingly popular as complementary and alternative medicines in combination with allopathic pharmaceuticals. Indeed, some plant products (e.g., *Harpagophytum procumbens* (Burch.) DC. Ex Meisn., commonly known as devil‘s claw extracts) are commonly sold at pharmacies globally, and it is no longer necessary to visit a traditional Muthi market to obtain products developed from them. However, a substantial portion of traditional plant use in South Africa does use plant materials obtained and prepared following traditional methods. Depending on the plant species used, a variety of different parts including roots, flowers, leaves, bulbs, and stems may be used medicinally, and the individual parts may have substantially different properties and uses [29]. Traditional beliefs have a deep influence within the majority of the ethnic cultures in South Africa and are particularly prevalent in rural communities [30]. Even in urban communities, a large portion of the South African population is reliant on traditional medicine as their primary mode of healthcare [31]. Indeed, that study postulated that the demand for traditional medicines in South Africa will increase in future years due to stress associated with urban lifestyles.

Despite their widespread use, there is a relative lack of information on the proper use and preservation of plant medicines. Medicinal plants are considered (often erroneously) to have fewer adverse effects [32] and are often more accessible and affordable than Western/allopathic medications [33]. A substantial number of South Africans (especially rural populations) are dependent on self-medication with plant-based medicines, and the involvement of the community in managing the use and preservation of plant species may result in successful strategies for sustainable use [34]. South African ethnobotanical literature has been relatively well recorded, although the medicinal properties of many species used traditionally are yet to be rigorously verified. There has been a substantial increase in studies screening and validating the use of South African traditional medicines in recent years, highlighting the potential of several species [35]. Of the therapeutic properties examined, the antibacterial activity of South African plants has received the most attention, although many species remain relatively neglected. Numerous plants have been reported to have antimicrobial activity, with a substantial recent increase in interest in this field. However, very few of those studies have specifically focussed on UTIs. Instead, screening against bacterial pathogens that cause gastrointestinal diseases [36, 37], skin disease [38, 39], or autoimmune diseases [40, 41] have received far greater attention. Notably, many of the same bacterial species screened in the other studies are also amongst the pathogenic causes of UTIs. Whilst the focus of those studies is not UTIs, they are included in this study as they were screened against the same bacterial species.

## 2. Materials and Methods

This study aimed to record and document the southern African medicinal plants that are used traditionally to treat UTIs. A variety of ethnobotanical books [42–46], as well as multiple peer reviewed journal articles, were consulted to compile this list. The online resources Google Scholar, PubMed, Scopus, and ScienceDirect were used to identify and access original scientific research studies. The following terms were used as filters and were searched for both alone and as combination: “Southern Africa,” “South African,” “Lesotho,” “Swaziland,” “Namibia,” “Botswana,” “Zimbabwe,” “Zambia,” “Mozambique,” “traditional medicinal plant,” “ethnobotany,” “urinary tract infection,” “UTI,” “bladder,” and “uropathogens.” The initial search aimed to document all of the plant species used in southern Africa to treat UTIs. Our study was nonbiased and did not favour the traditional knowledge of one ethnic group over others. Despite this, substantially more information was available about Zulu traditional medicine due to the prevalence of reports on that topic in the available literature. Whilst most of these species are native South African plants, introduced species were not excluded, where they had been incorporated into the traditional medicine systems of at least one South African ethnic group. Following the initial literature review, a further review was undertaken to identify the species that have been screened for their ability to inhibit one or more of the bacterial pathogens that cause UTIs.

### 2.1. Eligibility Criteria

Ethnobotanical books and peer reviewed journal articles were searched using the specific key words noted above. Published studies were identified and their abstracts were read to establish their relevance to this study. The full content of publications that were deemed relevant were then examined thoroughly to ensure that the eligibility criteria were met.

### 2.2. Inclusion Criteria

The following inclusion criteria for eligibility of the study were considered:

- Publications written in English and prior to April 2021 were used in this review.
- Our study was nonbiased and without any taxonomic preference.
- For the ethnobotanical survey (Table 1), only plant species that are recorded to treat UTIs are included. Any plants recorded to treat individual nonspecific symptoms were excluded unless it could be determined that they were specifically used to treat UTIs.
- For the biological activity studies presented in Table 2, only studies that screened against the major bacterial causes of UTIs were included, irrespective of whether the focus of the study was UTIs or the bacteria tested were selected because of their association with a different disease.
- Only studies screening against the common causes of UTIs were evaluated in this review, irrespective of their focus. For example, studies that screened southern African plants against *E. coli* were included in this study, even if their focus was on gastrointestinal diseases rather than UTIs.
- Ethnobotanical studies on the flora of southern African region included South Africa and those countries immediately surrounding it.

### 2.3. Exclusion Criteria

The following criteria were used to exclude some studies:

- Where name changes and families of plant species were encountered, particularly in older publications, websites such as The Plant List (http://www.theplantlist.org/) and South African National Biodiversity Institute (SANBI) (http://www.sanbi.org) were used to confirm species identification.
- Plant species that were recorded to treat generic symptoms of UTIs that are common to other illnesses, without specifically stating their use in treating UTIs, were excluded from this study.
- Studies that screened against bacteria that only cause UTIs secondary to other diseases were excluded. Therefore, studies screening South African plants for *S. aureus*, which only causes UTIs secondary to blood *S. aureus* infections, were not included in this study.
- Only screening studies that tested against bacterial pathogens were included in this study. Publications that screened South African plants for fungi, viruses, or protozoa were excluded.
- The use of introduced plant species were excluded from this study unless they are extensively used as part of southern African traditional medicine of at least one South African ethnic group.

### 2.4. Data Collection

A thorough literature search for publications on southern African medicinal plants used traditionally to treat UTIs was undertaken and is summarised in this study. Additionally, *in vivo* and *in vitro* biological screening of South African medicinal plants for bacterial pathogens that cause UTIs are summarised, regardless of the origin of the study. The following data was collected for each species:

- Species name, family name, and common name for each species recorded in the individual publications were collected
- Common names and the names used by different ethnic groups (where appropriate) were collected from individual publications and from the SANBI red list website
- The plant part used, method of preparation, and mode of administration were recorded where that information was provided

Microsoft Excel was used for statistical data analysis.

## 3. Results

### 3.1. Plants Used Traditionally to Treat Urinary Tract Infections

**Figure 1:**
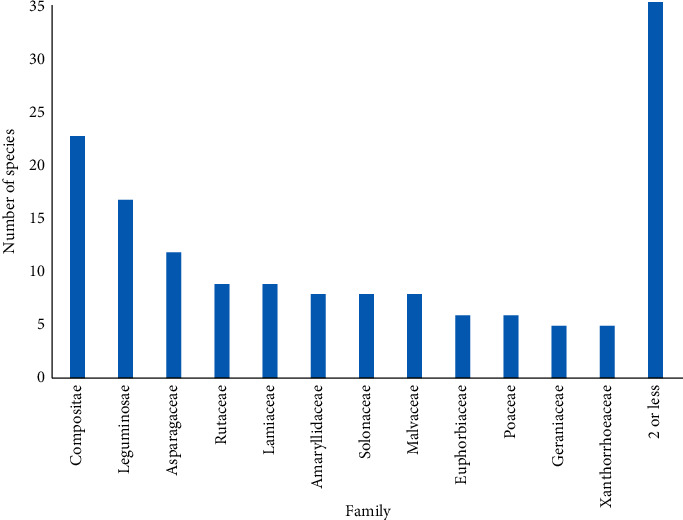
The number of species per plant family used to treat urinary tract infections. “2 or less” indicates families where there were ≤ two plant species represented.

Numerous South African plant species have been recorded to treat pathogenic diseases. This knowledge has traditionally been passed down from generation to generation by word of mouth, and some of this knowledge has now been recorded in ethnobotanical publications, although it is likely that substantial information is not yet readily available. A total of 153 plants from fifty-two families were recorded in the literature for the treatment of UTIs (Table 1). Out of the fifty-two families, Compositae had the greatest representation, with nineteen species reported as treatments for UTIs (Figure 1). Leguminosae were also commonly used as UTI therapies, with fourteen plant species reported [42, 43]. Asparagaceae, Rutaceae, and Lamiaceae were also well represented, with eight, six, and six species used to treat UTIs, respectively. Five species each of Amaryllidaceae, Solanaceae, and Malvaceae, as well as four species of both Euphorbiaceae and Poaceae, and three species of Geraniaceae and Xanthorrhoeaceae were also used for this purpose. A further twenty-four plant families were represented by two or fewer individual species. Of these, *Aptosimum procumbens* (Lehm.) Burch. ex Steud, *Arctopus echinatus* L., *Boophone disticha* (L.f.) Herb., *Bowiea volubilis* Harv*., Cardiospermum halicacabum* L., *Cissampelos capensis* L.f., *Galenia africana* L., *Helichrysum odoratissimum* (L.) Sweet, and *Zantedeschia albomaculata* (Hook.) Baill have been cited by several sources that also experimentally validated their use for the treatment of UTIs [47, 62, 64]. Approximately 47% of the plant species identified in our study were cited by multiple sources as traditional UTI therapies, indicating that screening and validation of those species should be prioritised.

**Figure 2:**
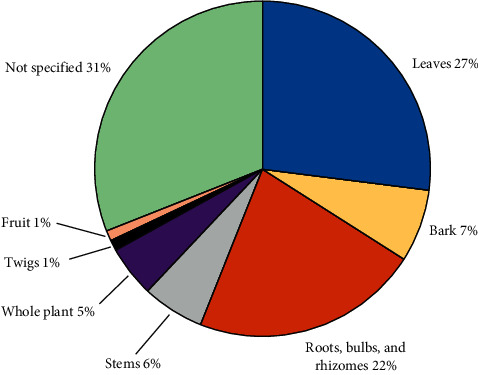
Frequency of use of different plant parts used to treat urinary tract infections in southern African traditional medicine.

The main plant parts used to prepare therapies to treat UTIs are leaves (27%), followed by roots, bulbs, and rhizomes (22%) (Figure 2). For 48 plant species (31%), the specific plant part used was not specified in the cited literature. For *Solanum capense* L. and *Pelargonium ramosissimum* Willd., both leaves and stems were used to treat UTIs, so both parts are recorded in Table 1 for that purpose [43, 58]. Fruits were found to be the least used parts as they are only available for short periods seasonally and may not be always readily available.

### 3.2. Dosage and Toxicity

Long term use of medicinal plants to treat diseases has resulted in the assumption that medicinal plants are nontoxic and safe for therapeutic use [39]. Of the plants specified for traditional use for UTIs (Table 1), none had their therapeutic dosage ranges recorded. It is noteworthy that several of these species have been reported to contain cardiac glycoside toxins (for example, *Bowiea volubilis* and *Acokanthera oppositifolia*) [70, 85, 86] and cucurbitacin (widely distributed in the Cucurbitaceae, Rubiaceae, Euphorbiaceae, and Cruciferae families), which have several adverse effects and toxicities [87]. Thus, further studies into the pharmacological and safety profiles of the majority of the plants listed in Table 1 are required to determine their safety for the treatment of UTIs. Indeed, a common trend noted was that information on dosage and toxicity is lacking, not only for the treatment of UTIs, but in the records listing South African plant use against many other diseases.

### 3.3. Scientific Studies on the Effects of Southern African Plants against Urinary Tract Infections

In a review of South African plants that have been studied for their antimicrobial properties, it was concluded that, before 2017, there had been no specific uropathogenic studies focusing on the antimicrobial activities of southern African medicinal plants [35]; however, pathogens such as *E. coli* were included in other screening studies. For example, *E. coli* is also associated with gastrointestinal diseases, and some strains of this pathogen cause diarrhoea [88]. It is therefore not surprising that studies screening South African plants against *E. coli* more frequently focus on its involvement in those diseases [73, 77, 82]. *Escherichia coli* is also a widely studied bacterium and is often included in studies performing screening against generic bacterial panels. Whilst these studies do not focus on the involvement of *E. coli* in UTIs, they have still been reported in this study as they demonstrate inhibitory activity against this bacterium, regardless of infection site. Similarly, *P. mirabilis* and *K. pneumoniae* are also associated with other diseases, including the autoimmune diseases, rheumatoid arthritis, and ankylosing spondylitis, respectively [40, 41]. Furthermore, *Pseudomonas aeruginosa* not only causes UTIs, but also has been associated with several other diseases [89]. Studies that screened southern African plant extracts for these bacteria were also included, regardless of the disease state that was the focus of the study.

A total of 85 plants used in southern African traditional medicine to treat UTIs have been tested for inhibitory activity against at least one UTI-causing bacterium. Not surprisingly, the inhibitory properties of southern African traditional medicine plants against *E. coli* were particularly well studied. Indeed, 82 species (96% of the total plant species screened) have previously been reported to inhibit *E. coli* growth at a noteworthy concentration. Notably, the majority of the plant species that have been screened against *E. coli* have also been screened against one or more other UTI-causing bacteria. Screening of southern African plants against *P. aeruginosa* has also received substantial attention, with 36 southern African plants reported to have noteworthy activity against this species. This is important as *P. aeruginosa* is resistant to many conventional antibiotics. Therefore, plant species with activity against this bacterium may be particularly promising, not only against UTIs, but also against other diseases in which *P. aeruginosa* causes pathogenesis, including multiple sclerosis [89] and cystic fibrosis [84]. Particularly, good *P. aeruginosa* inhibitory activity was reported for two *Terminalia* spp. (*T. phanerophlebia* and *T. sambesiaca*), with MICs of 80 and 60 *μ*g/mL, respectively [79]. *Proteus* spp. and *K. pneumoniae* were each screened against 14 southern African plant species. Nearly all of the plants screened against those bacteria were tested in two separate studies that used the same panels of plant species [40, 41]. Both of those studies screened a larger panel of South African medicinal plants than listed here, and only those plant species with appreciable activity are reported herein. Notably, we were only able to find reports of *S. saprophyticus* inhibitory activity for two closely related South African plants of the genus *Alchornea* (*A. cordifolia* and *A. laxiflora*) [74]. As this bacterium is responsible for approximately 6% of uncomplicated UTIs, screening of South African plants for this bacterium is a priority for future studies.

Notably, 46 of the bacterial screening studies reported herein did not test toxicity within the same study, and it is therefore not possible to determine therapeutic indexes. Therefore, whilst the plant species examined in those studies may have noteworthy antibacterial activity, it is not possible to comment on their safety and therefore their potential as therapies to treat UTIs. Of the plants that were screened for toxicity alongside antibacterial activity, LC_50_ values that indicate a lack of toxicity were reported for 28 plant species. Of concern, 11 plant species (*A. laxiflora, Bolusanthus speciosus, Calpurnia aurea, Cremaspora triflora, E. croceum, Heteromorpha arborescens, Hypericum roeperianum, Maesa lanceolata, Morus mesozygia, Pittosporum viridiflorum,* and *Tulbaghia violacea*) were reported to have appreciable toxicity, and therefore caution is recommended for their use. Of particular concern, LC_50_ values of 2–14 *μ*g/mL were reported for *C. triflora, E. croceum,* and *M. lanceolata.* Given the MIC values of these plant species against *E. coli*, the therapeutic indexes (as low as 0.008) can be calculated, indicating that these extracts are extremely unsafe for therapeutic use as the extracts are toxic at ∼1% of the concentration required to achieve the therapeutic effect. However, it is noteworthy that all of these low LC_50_ values in Vero cells were determined in a single study [77]. That study also reported low LC_50_ values (lower than 100 *μ*g/mL) for every plant species that was tested in that study, and therefore the results may be an anomaly of that study. The toxicity of these plant species needs to be verified in future studies to evaluate their safety for therapeutic use.

## 4. Discussion and Conclusion

Urinary tract infections are one of the most widespread classes of infectious diseases globally, yet the development of new therapies to treat these infections remains relatively neglected. Whilst UTIs cause discomfort, they rarely cause mortality or serious morbidity except in immune-compromised individuals. It is likely that the relative lack of severity of these infections may contribute to the low number of studies into the effects of southern African plants specifically targeted at bacteria that cause these infections. Indeed, most of these studies have occurred in the most recent 15-year period. Interestingly, the increase in research in this field coincides with increases in the incidence of antibiotic resistance. Several studies have already screened some South African plants for the ability to inhibit UTI-causing bacteria. Indeed, Table 2 summarises studies screening 85 plant species against UTI-causative bacteria.

Surprisingly, despite 153 plant species identified with documented uses to treat UTIs, only 85 species have been reported to have noteworthy inhibitory activity against the main UTI-causative bacteria species. Furthermore, most of the tested plants were not selected for screening based on their traditional use to treat UTIs. Instead, in many cases, the plant species were screened against the bacteria based on their involvement in other diseases. *Escherichia coli* is a common gastrointestinal bacterium, and several studies screened plant extracts against this bacterium to examine its potential to treat diarrhoea. The studies described in this review focussed on the main bacterial species that can cause UTIs (*E. coli, K. pneumoniae, P. aeruginosa, P. mirabilis,* and *S. saprophyticus*). Combined, these bacteria account for >90% of the cases of uncomplicated UTIs, highlighting their importance to those studies [11]. However, for these pathogens, we were only able to locate a single study that tested South African plants against *S. saprophyticus* [74], and future studies screening plants against this bacterium are required. Furthermore, other bacterial species are also responsible for a significant proportion of UTIs. In particular, unspecified *Enterococcus* spp. account for approximately 6% and 11% of uncomplicated and complicated UTIs, respectively [11], and therefore future studies screening South African plants against these pathogens are warranted. Furthermore, our review focussed on the bacterial causes of UTIs as they account for nearly all reported cases. However, *C. albicans*, as well as some viruses and protozoa, may also cause UTIs and should not be neglected in future studies. Further studies are required to screen those species against the other major causes of UTIs. Additionally, all the previous studies have screened plant species against UTI-causative bacteria that are susceptible to conventional antibiotic therapies. To date, screening plants against antibiotic-resistant bacterial strains has been largely neglected. As there have been substantial increases in the prevalence of antibiotic-resistant bacterial pathogens in recent years, it is important that the plants identified herein are also screened against resistant bacterial strains to further evaluate the potential in clinical environments. Furthermore, when examining antimicrobial efficacy, it must be noted that UTIs are very often biofilm-borne, and this aspect has been neglected in the screening. Only one study [78] focussed on 14 plant species utilising bacterial communication systems via antiquorum sensing signalling and in biofilm studies. Clearly, this area remains untapped, and there is a possibility for plant species to not only act on planktonic bacterial cells, but also inhibit or prevent biofilm formation.

It appears that plant species selection for several studies was based more on plant availability rather than ethnobotanical use. Another aspect of the previous studies is that the plant part tested does not always correlate with the part traditionally used. It is likely that availability may also have been a significant factor in selection of plant part in those studies. However, the chemistry may differ substantially between different plant parts, and they may therefore induce completely different biological activities. Where possible, screening and evaluation studies should test the plant part used traditionally, as well as an approximation of how it was processed for use. These factors may have significant impacts on the toxicity of the preparation. Future studies testing an approximation of the traditional plant preparations are therefore required to validate the traditional use of these plants to treat UTIs.

To be useful in the treatment of UTIs, an extract (or purified compound derived from an extract) must have relatively low toxicity. This is particularly true for the treatment of recurrent infections. Surprisingly, many of the plant species screened for inhibitory activity against UTI-causative bacteria were not tested for toxicity in the same studies. This may be because many of these plants have been used in traditional healing systems for hundreds of years without reported toxicity and are therefore assumed to be safe. However, some plant species are prepared by different methods to test bioactivity from the preparation method used by traditional medicine practitioners. Different preparation methods may dramatically alter the phytochemical composition of different preparations and therefore may also affect their toxicity profiles. Indeed, several studies reported toxic, carcinogenic, and mutagenic effects for extracts prepared from plants traditionally used as medicines [33]. Several of the studies highlighted in this review have also investigated the toxicity of southern African plant species traditionally used to treat UTIs [40, 41, 71, 73, 77, 80]. Whilst most of those studies reported that the plant extracts were nontoxic, one study reported very low LC_50_ values against Vero cells for several plant species, indicating that those species may not be safe to use medicinally. However, the results of that study may be erroneous as all the plant species tested had low LC_50_, and verification of these results is required. Of further note, several different toxicity assays (human cell lines, Vero monkey kidney cells, *Artemia* nauplii assays) were used to screen the plant species in different studies, making comparisons difficult between studies. Ideally, toxicity studies should incorporate more than one toxicity assay to allow for better comparisons between studies.

Ethnobotanical records have already identified several promising plant species used in traditional South African medicinal systems to treat UTIs, and several of those species (as well as multiple species for which a traditional use against UTIs was not recorded) have already been tested against one or more UTI-causative bacteria. Further research is required to screen all identified species against each of the UTI-causative bacteria (rather than just one or two of them) and against antibiotic-resistant bacterial strains that are becoming increasingly common. Additionally, all previous studies have tested the plant extracts *in vitro*. As considerably different effects may be seen *in vivo* due to bioavailability differences, studies in animal models are also required.

Overall, there is evidence that southern African medicinal plants have potential to treat UTIs and, with further in-depth analysis, could be the new alternative to cranberry juice which is internationally recognised as a safe natural alternative for treating infections of the urinary tract.

## Figures and Tables

**Table 1:** Southern African plants traditionally used to treat urinary tract infections.

Plant species	Family	Common name	Part of plant used	Uses	References
*Acacia sieberiana* var. *woodii* (Burtt Davy) Keay & Brenan	Leguminosae	Paperbark thorn (Eng); papierbasdoring (Afr)	Bark and roots	Urinary tract ailments	[46]
*Acokanthera oppositifolia* (Lam.) Codd	Apocynaceae	Inhlungunyembe (Zulu)	Not specified	Urinary tract infection	[43]
*Afroaster hispida* (Thunb.) J.C.Manning & Goldblatt	Asteraceae	Udlutshana (Zulu)	Not specified	Urinary ailments	[47]
*Agathosma betulina* (P.J.Bergius) Pillans	Rutaceae	Buchu (Eng); boegoe (Afr); bocho (Sotho)	Leaves	Bladder and kidney ailments	[45, 48, 49]
*Agathosma capensis* (L.) Dummer	Rutaceae	Spicy buchu (Eng); anysboegoe, steenbokboegoe (Afr)	Leaves	Urinary ailments	[50]
*Agathosma serratifolia* (Curtis) Spreeth	Rutaceae	Langblaarboegoe, kloofboegoe (Afr); long buchu (Eng)	Leaves	Bladder and kidney ailments	[45]
*Albizia adianthifolia* (Schum.) W.Wight	Leguminosae	Isiyengelele, usolo, umgadankawu (Zulu)	Not specified	Urinary tract infection	[47]
*Aloe ferox* Mill.	Xanthorrhoeaceae	Bitteraalwyn (Afr); bitter aloe (Eng)	Leaves, roots, and stems	Kidney and bladder ailments	[51]
*Aloe zebrina* Baker	Xanthorrhoeaceae	Sebra-aalwyn (Afr); zebra aloe (Eng)	Leaves	Urinary and bladder ailments	[46]
*Antiphiona pinnatisecta* (S.Moore) Merxm.	Compositae	Unknown	Roots	Bladder ailments	[46]
*Antizoma angustifolia* (Burch.) Miers ex Harv.	Menispermaceae	Maagbitterwortel (Afr)	Roots	Kidney and bladder ailments	[52]
*Aptosimum procumbens* (Lehm.) Burch. ex Steud	Scrophulariaceae	Brandbos (Afr)	Leaves	Bladder ailments	[42–44]
*Arctopus echinatus* L.	Apiaceae	Platdoring (Afr)	Roots	Bladder ailments	[42–44, 53]
*Artemisia afra* Jacq. ex Willd.	Compositae	Alsem (Afr); African wormwood (Eng); mhlonyana (Zulu)	Leaves, roots, and stems	Bladder and kidney ailments	[49, 51]
*Asparagus africanus* Lam.	Asparagaceae	Bush asparagus, African asparagus (Eng); katdoring (Afr); isigobo (Zulu)	Not specified	Bladder and kidney ailments	[46]
*Asparagus asparagoides* (L.) Druce	Asparagaceae	Makholela (Sotho)	Roots	Urinary tract infection	[48]
*Aster bakerianus* Burtt Davy ex C.A.Sm.	Compositae	Idlutshane, uhloshana (Zulu)	Leaves	Urinary tract infection	[43]
*Baccharoides adoensis* var. *kotschyana* (Sch.Bip. ex Walp.)	Compositae	Innyathelo (Zulu)	Stems and leaves	Urinary tract infection	[43]
*Ballota africana* (L.) Benth.	Lamiaceae	Kattekruid (Afr)	Leaves	Bladder and kidney ailments	[50]
*Berkheya Setifera* DC.	Compositae	Ikhakhasi, umalumvuba (Zulu); leleme-la-khomo (Sotho)	Root	Urinary and kidney ailments	[47, 54]
*Bolusanthus speciosus* (Bolus) Harms	Leguminosae	Umholo (Zulu); tree wisteria (Eng); vanwykshout (Afr)	Barks, leaves, and stems	Kidney ailments	[55]
*Boophone disticha* (L.f.) Herb.	Amaryllidaceae	Gifbol (Afr)	Bulb	Bladder ailments	[42–44]
*Bowiea volubilis* Harv.	Asparagaceae	Climbing onion (Eng); knolklimop (Afr); iguleni (Zulu)	Bulb	Bladder ailments	[43, 48]
*Brachylaena discolor* DC.	Compositae	Bosvaalbos (Afr); ipahla (Zulu)	Leaves	Urinary ailments	[43]
*Bulbine abyssinica* A.Rich	Xanthorrhoeaceae	Wildekopieva (Afr)	Leaves	Bladder ailments	[56, 57]
*Bulbine latifolia* (L.f.) Spreng.	Xanthorrhoeaceae	Red carrot (Eng); rooiwortel (Afr)	Root	Bladder and kidney ailments	[48, 57]
*Cardiospermum halicacabum* L.	Sapindaceae	Balloon vine (Eng); blaasklimop (Afr)	Stems and leaves	Bladder ailments	[43, 46, 58]
*Carica papaya* L.	Caricaceae	Papaya, pawpaw (Eng)	Not specified	Bladder ailments	[42]
*Cenchrus ciliaris* L.	Poaceae	Buffalograss (Eng); bloubuffelgras (Afr); idungamuzi (Zulu)	Roots	Urinary tract infection	[43, 59, 60]
*Centaurea benedicta* (L.) L.	Compositae	Karmedik (Afr)	Not specified	Urinary and kidney ailments	[50]
*Chironia baccifera* L.	Gentianaceae	Bitterbos, sarsaparilla (Afr)	Not specified	Urinary ailments	[50]
*Chrysanthemoides monilifera* (L.) Norl.	Compositae	Bietou, bessiebos (Afr)	Whole plant	Urinary and kidney ailments	[50]
*Cissampelos capensis* L.f.	Menispermaceae	David's root (Eng); gifhondjie, dawidjieswortel (Afr)	Roots and leaves	Bladder ailments	[42–44, 46]
*Cleome gynandra* L.	Cleomaceae	African cabbage, spiderwisp (Eng); snotterbelletjie (Afr)	Not specified	Bladder ailments	[42]
*Cliffortia odorata* L.f.	Rosaceae	Wildewingerd (Afr); wild vine (Eng)	Leaves	Bladder ailments	[48]
*Clivia miniata* (Lindl.) Bosse	Amaryllidaceae	Benediction lily (Eng); boslelie (Afr); umayime (Zulu)	Bulb	Urinary ailments	[43]
*Coix lacryma-jobi* L.	Poaceae	Job's tears, adlay (Eng)	Not specified	Bladder ailments	[42]
*Combretum kraussii* Hochst.	Combretaceae	Umduba, umdubu omhlophe, umdubo wamanzi (Zulu)	Not specified	Urinary and bladder ailments	[47]
*Commelina africana* L.	Commelinaceae	Idangabane (Zulu); khopo (Sotho)	Root	Bladder ailments	[43]
*Conostomium natalense* (Hochst.) Bremek.	Rubiaceae	Wild pentas (Eng); umbophe, ungcolosi (Zulu)	Not specified	Urinary ailments	[61]
*Conyza scabrida* DC.	Compositae	Oondbos, bakbos, paddabos (Afr)	Leaves	Bladder infection	[51]
*Crinum macowanii* Baker	Amaryllidaceae	River fly (Eng); umduze (Zulu)	Bulb	Urinary ailments	[43]
*Crinum moorei* Hook. f.	Amaryllidaceae	Umduze (Zulu)	Bulb	Urinary ailments	[43]
*Crossyne guttata* (L.) D.Müll.-Doblies & U.Müll.-Doblies	Amaryllidaceae	Gifbol (Afr)	Bulb	Bladder ailments	[42–44]
*Cryptolepis oblongifolia* (Meisn.) Schltr.	Apocynaceae	Bokhoring, melkbos (Afr); mukangaza (Sotho)	Not specified	Bladder ailments	[42]
*Cussonia paniculata* Eckl. & Zeyh.	Araliaceae	Motsetse (Sotho)	Not specified	Kidney and bladder ailment	[54, 62]
*Cyathula achyranthoides* (Kunth) Moq.	Amaranthaceae	Unknown	Not specified	Bladder ailments	[63]
*Cynodon dactylon* (L.) Pers.	Poaceae	Dog's tooth (Eng); krookgras (Afr)	Rhizome	Urinary ailments	[46]
*Datura stramonium* L.	Solanaceae	Thornapple, jimsonweed, devil's snare (Eng)	Leaves	Bladder ailments	[42]
*Dicoma capensis* Less.	Compositae	Karmedik, wilde karmedik (Afr)	Leaves	Bladder and kidney ailments	[44, 45, 56, 57]
*Diosma oppositifolia* L.	Rutaceae	Buchu, Bitter buchu (Eng)	Leaves	Bladder and kidney ailments	[48]
*Diosma prama* I.Williams	Rutaceae	Steenbokkboegoe (Afr)	Not specified	Urinary and kidney ailments	[50]
*Diospyros mespiliformis* Hochst. ex A.DC	Ebenaceae	Ikhambi lesduli, umazambezi (Zulu)	Not specified	Urinary ailments	[47]
*Dipcadi gracillimum Baker*	Asparagaceae	Oumasegroottoon (Afr)	Not specified	Bladder ailments	[63]
*Dipcadi viride* (L.) Moench	Asparagaceae	Ugibizisila, uguleni, umakhweyana (Zulu)	Not specified	Urinary tract infection	[47]
*Dipcadi gracillimum* Baker	Asparagaceae	Oumasegroottoon (Afr)	Not specified	Urinary ailments	[64]
*Dodonaea viscosa* (L.) Jacq.	Sapindaceae	Basterolen, sandolien, ysterbos (Afr); sand olive (Eng)	Leaves	Bladder and kidney ailments	[5, 50, 51]
*Drimia elata* Jacq.	Asparagaceae	Satin squill (Eng); brandui, jeukbol (Afr); umqumbu (Zulu)	Not specified	Urinary ailments	[44]
*Dysphania ambrosioides* (L.) Mosyakin & Clemants	Amaranthaceae	Ikhambi leslumo, uzansikwesibaya (Zulu)	Not specified	Urinary ailments	[47]
*Elytropappus rhinocerotis* (L.f.) Less.	Asteraceae	Renosterbos (Afr)	Leaves	Bladder and kidney disorders	[49]
*Elymus repens* (L.) Gould	Poaceae	Couch grass (Eng)	Not specified	Bladder ailments	[42]
*Empleurum unicapsulare* (L.f.) Skeels	Rutaceae	Bergboegoe, langblaarboegoe (Afr)	Whole plant	Urinary and kidney ailments	[50]
*Eriocephalus punctulatus* DC.	Compositae	Kapokbos (Afr)	Whole plant	Urinary and kidney ailments	[50]
*Eriosema distinctum* N.E.Br.	Leguminosae	Ubangalala omkhulu (Zulu)	Roots	Urinary ailments	[43]
*Erythrina caffra* Thunb.	Leguminosae	Coral tree (Eng); kaffirboom (Afr); umsinsi (Zulu)	Leaves	Urinary ailments	[43]
*Erythrina lysistemon* Hutch.	Leguminosae	Umsinsi (Zulu)	Leaves	Bladder ailments	[47]
*Euclea natalensis* A.DC.	Ebenaceae	IsiZimane, umshekisane (Zulu)	Bark	Urinary tract infection	[47, 65]
*Euclea undulata* Thunb.	Ebenaceae	Ghwarrie, ghwarrieboom, ghwarriebos (Afr)	Whole plant	Urinary and kidney ailments	[43, 50]
*Eucomis autumnalis* (Mill.) Chitt	Asparagaceae	Mathethebane (Sotho)	Tubular roots	Urinary ailments	[58]
*Euphorbia milii* Des Moul.	Euphorbiaceae	Crown of thorns (Eng)	Not specified	Bladder ailments	[64, 66]
*Euphorbia tithymaloides* L.	Euphorbiaceae	Redbird flower (Eng)	Not specified	Bladder ailments	[64, 66]
*Exomis microphylla* (Thunb.) Aellen	Amaranthaceae	Hondepisbos, hondebossie (Afr)	Whole plant	Urinary ailments	[50]
*Faidherbia albida* (Delile) A.Chev.	Leguminosae	Anatree (Eng); anaboom (Afr)	Bark	Bladder ailments	[46]
*Foeniculum vulgare* Mill.	Apiaceae	Vinkel, makuinkel, soetvinkel (Afr)	Whole plant	Urinary ailments	[50]
*Galenia africana* L.	Aizoaceae	Kraalbos (Afr)	Leaves	Bladder ailments	[42–44]
*Galium tomentosum* Thunb.	Rubiaceae	Red storm (Eng); rooistorm, doodlief (Afr)	Root	Bladder ailments	[48, 56]
*Geranium incanum* Burm.f.	Geraniaceae	Carpet geranium (Eng); horlosie, bergtee, vrouetee (Afr); tlako (Sotho)	Not specified	Bladder ailments	[56, 64]
*Grewia caffra* Meisn.	Malvaceae	Upata, iphatha (Zulu)	Roots, bark	Bladder ailment	[67]
*Grewia occidentalis* L.	Malvaceae	Cross berry (Eng); iklolo, imahlehle (Zulu)	Roots	Bladder ailments	[43]
*Grewia robusta* Burch.	Malvaceae	Bokbos (Afr)	Whole plant	Urinary ailments	[50]
*Gunnera perpensa* L.	Gunneraceae	Ugobho, izibu (Zulu)	Not specified	Bladder ailments	[47]
*Helichrysum crispum* (L.) D. Don	Compositae	Kooigoed (Afr)	Leaf	Bladder and kidney ailments	[49]
*Helichrysum odoratissimum* (L.) Sweet	Compositae	Phefo (Sotho); kooigoed, kooibos (Afr)	Leaves, roots, and stems	Bladder ailments	[50, 56]
*Helichrysum patulum* (L.) D.Don	Compositae	Honey everlasting (Eng); kooigoed (Afr); impepho (Zulu)	Not specified	Bladder ailments	[42]
*Hibiscus pusillus* Thunb.	Malvaceae	Blaasbossie (Afr)	Whole plant	Urinary ailments	[50]
*Hibiscus mastersianus* Hiern	Malvaceae	Monarch rosemallow (Eng)	Stems and Leaves	Urinary ailments	[46]
*Hibiscus pedunculatus* L.f.	Malvaceae	Pink mallow (Eng); indola ebomvu (Zulu)	Leaves	Urinary ailments	[43]
*Hoslundia opposita* Vahl	Lamiaceae	Bird gooseberry (Eng); uyaweyawe (Zulu)	Not specified	Urinary ailments	[46]
*Hypoxis hemerocallidea* Fisch., C.A.Mey. & Avé-Lall.	Hypoxidaceae	Inkanfe (Zulu); yellow star (Eng)	Not specified	Bladder ailments	[47, 50]
*Hypoxis rigidula* Baker	Hypoxidaceae	Ilabatheka, inkomfe, umhungulo (Zulu); African potato (Eng)	Not specified	Bladder ailments	[47, 50]
*Indigofera cassioides* DC.	Leguminosae	Unknown	Not specified	Bladder ailments	[42]
*Ipomoea pes-caprae* (L.) R.Br.	Convolvulaceae	Beach morning glory, goat's foot (Eng); strandpatat (Afr)	Not specified	Bladder ailments	[42]
*Jasminum abyssinicum* Hochst. ex DC.	Oleaceae	Mthundangazi	Roots	Bladder ailments	[68]
*Kedrostis capensis* A. Meeuse	Cucurbitaceae	Sesepa sa linoha (Sotho)	Tubular roots and leaves	Urinary tract infection	[69]
*Ledebouria marginata* (Baker) Jessop	Asparagaceae	Bokhoe (Sotho)	Root bulb	Urinary tract infection	[70]
*Leonotis leonurus* (L.) R.Br.	Lamiaceae	Klip dagga, wild dagga (Afr)	Stems with leaves and flowers	Bladder and kidney disorders,	[49]
*Lessertia frutescens* (L.) Goldblatt & J.C.Manning	Leguminosae	Kalkoenblom, keurtjie (Afr)	Leaves	Kidney and urinary ailments	[50, 56]
*Matricaria chamomilla* L.	Compositae	Chamomile (Eng)	Not specified	Bladder ailments	[42]
*Melianthus pectinatus* Harv.	Melianthaceae	Kriekiebos, lidjiebos (Afr)	Root	Urinary tract infection	[56]
*Mentha longifolia* (L.) L.	Lamiaceae	Wild mint (Eng); ufuthana lomhlanga (Zulu)	Leaves	Bladder and kidney ailments	[49, 52, 68]
*Merwilla plumbea* (Lindl.) Speta	Asparagaceae	Inguduza, untabosizi, untangana zibomvana (Zulu)	Not specified	Bladder ailments	[47, 50]
*Mesembryanthemum cordifolium* L.f.	Aizoaceae	Ibohlololo (Zulu)	Leaves	Bladder ailment	[44, 47, 53]
*Mesembryanthemum crystallinum* L.	Aizoaceae	Common ice plant, crystalline ice plant (Eng); soutslaai, volstruisslaai (Afr)	Not specified	Bladder ailments	[42]
*Mikania capensis* DC.	Compositae	Umdlonzo, umhlozo (Zulu)	Leaves	Urinary ailments	[42, 43]
*Millettia oblata* Dunn	Leguminosae	Unknown	Roots	Bladder ailments	[42]
*Notobubon galbanum* (L.) Magee	Apiaceae	Blister bush (Eng); bergseldery (Afr)	Not specified	Kidney and bladder ailment	[42, 52]
*Nymphaea nouchali* Burm.f.	Nymphaeaceae	Blue water lily (Eng); blouwaterlelie (Afr); izubu, iziba, ugobho (Zulu)	Leaves	Urinary tract infection	[46, 47, 49]
*Ocimum americanum* L.	Lamiaceae	Hoary basil (Eng); wilde basielkruid (Afr)	Not specified	Urinary tract infection	[46]
*Ocotea bullata* (Burch.) E. Meyer in Drege	Lauraceae	Stinkwood, laurel wood (Eng); umnukani, umhlungulu (Zulu)	Bark	Urinary ailments	[43, 58]
*Olea europaea* L.	Oleaceae	Wild olive (Eng); olyfhout, olienhout (Afr); isadlulambezo (Zulu)	Roots and bark	Urinary tract infection	[43, 46]
*Oncoba spinosa* Forssk.	Salicaceae	Snuff-box tree (Eng); snuifkalbassie (Afr)	Not specified	Bladder ailments	[42]
*Opuntia ficus-indica* (L.) Mill.	Cactaceae	Foie e kubedu (Sepedi); mudoro (Venda)	Roots	Urinary ailments	[63]
*Pedalium murex* L.	Pedaliaceae	Large caltrops (Eng)	Leaves	Bladder ailments	[42]
*Pegolettia baccharidifolia* Less.	Compositae	Ghwarrieson, heuningdou (Afr)	Leaves and twigs	Bladder and kidney ailments	[44, 45]
*Pelargonium grossularioides* (L.) L'Hér.	Geraniaceae	Rooirabas (Afr); gooseberry-leaved *Pelargonium* (Eng)	Leaves and stems	Urinary tract infection	[51, 68]
*Pelargonium hypoleucum* Turcz.	Geraniaceae	Rooirabas (Afr)	Roots	Urinary tract infection	[56]
*Pelargonium ramosissimum* Willd.	Geraniaceae	Dassieboegoe, dassiebos (Afr)	Leaves and stems	Bladder and kidney ailments	[51, 68]
*Pentanisia prunelloides* (Klotzsch) Walp.	Rubiaceae	Sooibrandbossie (Afr); icimamlilo (Zulu)	Roots	Bladder and kidney ailments	[43]
*Petroselinum crispum* (Mill.) Nyman ex A.W.Hill.	Apiaceae	Pietersielie (Afr); parsley (Eng)	Leaves	Bladder ailments	[49, 66]
*Phytolacca heptandra* Retz.	Phytolaccaceae	Inkbessie (Afr); ingubivumile (Zulu)	Not specified	Urinary ailments	[43]
*Portulaca quadrifida* L.	Portulacaceae	Pigweed, wild purslane (Eng); kanniedood (Afr)	Not specified	Bladder and kidney ailments	[42, 46]
*Prunus persica* (L.) Batsch	Rosaceae	Peach (Eng)	Leaves	Bladder ailments	[42]
*Ranunculus multifidus* Forssk.	Ranunculaceae	Botterblom, brandblare (Afr); uxhaphozi (Zulu)	Leaves	Urinary ailments	[43]
*Rhamnus prinoides* L'Hér.	Rhamnaceae	Mofifi (Sotho)	Root	Kidney and bladder ailment	[54]
*Rhoicissus tridentata* (L.f.) Wild & R.B.Drumm.	Vitaceae	Wild/bitter grape (Eng); bobbejaantou, wildedruif (Afr); isinwazi (Zulu)	Stems	Urinary ailments	[43, 64]
*Rhynchosia caribaea* (Jacq.) DC.	Leguminosae	Snoutbean (Eng); rankboontjie (Afr); isihlahlasenqomfi (Zulu)	Roots	Urinary ailments	[46]
*Rhynchosia minima* (L.) DC.	Leguminosae	Least snoutbean, burn-mouth-vine (Eng)	Roots	Bladder ailments	[46]
*Rhynchosia sublobata* (Schum.) Meikle	Leguminosae	Twiner of barren ground (Eng)	Roots	Bladder ailments	[46]
*Ricinus communis* L.	Euphorbiaceae	Olieboom, olieblaar (Afr)	Leaves	Urinary and kidney ailments	[47, 50]
*Rotheca hirsuta* (Hochst.) R.Fern	Lamiaceae	Butterfly bush, wild violet (Eng); umathanjana, lusikisiki (Zulu)	Not specified	Urinary ailments	[64]
*Ruta graveolens* L.	Rutaceae	Wynruit (Afr)	Leaves	Urinary tract infection and bladder ailments	[49, 56]
*Salix woodii* Seemen	Salicaceae	Wild willow (Eng)	Bark	Urinary ailments	[69]
*Salix mucronata* Thunb.	Salicaceae	Cape willow (Eng); vaalwilger (Afr)	Bark	Bladder ailments	[46]
*Salvadora persica* L.	Salvadoraceae	Toothbrush tree, real mustard tree (Eng); kerriebos (Afr)	Roots	Urinary tract infection	[46]
*Salvia microphylla* Kunth	Lamiaceae	Baby sage, Graham's sage, blackcurrant sage (Eng)	Roots	Urinary ailments	[50]
*Scadoxus puniceus* (L.) Friis & Nordal	Amaryllidaceae	Idumbe likahloyile, uhloyile, umphompo (Zulu)	Not specified	Urinary ailments	[47]
*Solanum aculeastrum* Dunal	Solanaceae	Gifappel (Afr); umthuma, untumane (Zulu)	Fruit	Urinary tract infection and kidney ailments	[47, 48]
*Sutherlandia frutescens* (L.) R.Br.	Fabaceae	Keurtjies, kankerbossie (Afr)	Leaves and stems	Bladder and kidney ailments	[49]
*Tarchonanthus* camphoratus L.	Compositae	Camphor bush (Eng); wilde kanferbos (Afr); igceba elimhlope (Zulu)	Not specified	Urinary ailments	[64]
*Teucrium trifidum* Retz.	Lamiaceae	Katjiedriebaar (Afr)	Leaves	Bladder ailments	[56, 57]
*Thesium hystrix* A.W.Hill	Santalaceae	Kleinswartstorm (Afr)	Roots	Bladder ailments	[42]
*Tragia meyeriana* Müll.Arg.	Euphorbiaceae	Stinging nettle (Eng); ubangalala, imbabazane (Zulu)	Not specified	Bladder ailments	[42, 43]
*Tragia rupestris* Sond.	Euphorbiaceae	Ubangalala, imbabazane (Zulu)	Roots	Bladder ailments	[43]
*Trichilia emetica* Vahl	Meliaceae	Red rash (Eng); basteressenhout (Afr); ixolo, umkhuhlu (Zulu)	Bark	Kidney ailments	[43]
*Trifolium africanum* Ser.	Leguminosae	Erasmus clover, wild clover (Eng); wildeklawer (Afr); moqoiqoi, moqophi (Sesotho)	Not specified	Bladder ailments	[63]
*Typha capensis* (Rohrb.) N.E.Br.	Typhaceae	Papkuil (Afr)	Not specified	Bladder and kidney ailments	[46]
*Urtica urens* L.	Urticaceae	Small nettle (Eng); dog nettle Eng)	Bark	Bladder pains	[58]
*Warburgia salutaris* (G.Bertol.) Chiov.	Canellaceae	Isibaha (Zulu)	Leaves	Urethral ailments	[67]
*Withania somnifera* (L.) Dunal	Solanaceae	Winter cherry (Eng); geneesblaar (Afr)	Roots	Bladder ailments	[46]
*Xanthium strumarium* L.	Compositae	Kankerroos (Afr)	Not specified	Bladder ailments	[42]
*Xysmalobium undulatum* (L.) W.T.Aiton	Apocynaceae	Wild cotton (Eng); melkbos (Afr)	Not specified	Bladder ailments	[46]
*Zantedeschia albomaculata* (Hook.) Baill.	Araceae	Arum lilies, calla lilies, pig lily (Eng.); varkblom (Afr); mohalalitoe (Sotho)	Not specified	Urinary ailments	[54, 62–64]
*Zea mays* L.	Poaceae	Corn (Eng); umbila (Zulu)	Not specified	Bladder ailments	[42]

**Table 2:** Plant species with noteworthy activity that have been tested against urinary tract bacterial pathogens.

Plant species	Plant part used	Pathogens screened	MIC values	Toxicity evaluation	References
*Acacia karoo* Hayne	Leaves	*E. coli*	**(**M) 414 *μ*g/mL; (W) 458 *μ*g/mL	Non-toxic in *Artemia* lethality assay	[71]

*Acacia nicolitica* (L.) Delile	Root and bark	*E. coli*	Root and bark: (E) 780 *μ*g/mL; (W) 6250 *μ*g/mL	Not determined	[72]

*Acacia sieberiana* DC.	Root and bark	*E. coli*	Root and bark: (E) 92–780 *μ*g/mL; (W) 1560 *μ*g/mL	Not determined	[72]
*Agathosma betulina* (Berg.) Pillans	Leaves	*E. coli*	(W) > 8000 *μ*g/mL	Not toxic in human epithelial kidney cells	[73]
		*K. pneumoniae*	(M) 1876 *μ*g/mL; (W) 2387 *μ*g/mL	Non-toxic in *Artemia* lethality assay	[41]
		*P. mirabilis*	(M) 878 *μ*g/mL		[40]

*Alchornea cordifolia* (Schumach. And Thonn.) Müll. Arg.	Leaves, stem	*E. coli*	Leaves: (M) 63 *μ*g/mL; (E) 63 *μ*g/mL	Not determined	[74]
			Stem (M) 63 *μ*g/mL; (E) 63 *μ*g/mL		
		*K. pneumoniae*	Leaves: (M) 125 *μ*g/mL; (E) 125 *μ*g/mL		
			Stem (M) 125 *μ*g/mL; (E) 125 *μ*g/mL		
		*P. mirabilis*	Leaves: (M) 125 *μ*g/mL; (E) 125 *μ*g/mL		
			Stem (M) 125 *μ*g/mL; (E) 250 *μ*g/mL		
		*S. saprophyticus*	Leaves: (M) 63 *μ*g/mL; (E) 63 *μ*g/mL		
			Stem (M) 63 *μ*g/mL; (E) 63 *μ*g/mL		

*Alchornea laxiflora* (Benth.) Pax & Hoffm.	Leaves, roots, stem	*E. coli*	Leaves: (M) 125 *μ*g/mL; (E) 125 *μ*g/mL	LC_50_ = 100–140 *μ*g/mL in HeLa cells	[74]
			Roots: (M) 500 *μ*g/mL; (E) 500 *μ*g/mL		
			Stem: (M) 500 *μ*g/mL; (E) 250 *μ*g/mL		
		*K. pneumoniae*	Leaves: (M) 63 *μ*g/mL; (E) 63 *μ*g/mL		
			Roots: (M) 125 *μ*g/mL; (E) 125 *μ*g/mL		
			Stem: (M) 500 *μ*g/mL; (E) 500 *μ*g/mL		
		*P. mirabilis*	Leaves: (M) 8000 *μ*g/mL; (E) 2000 *μ*g/mL		
			Roots: (M) 250 *μ*g/mL; (E) 250 *μ*g/mL		
			Stem: (M) 4000 *μ*g/mL; (E) 4000 *μ*g/mL		
		*S. saprophyticus*	Leaves: (M) 63 *μ*g/mL; (E) 63 *μ*g/mL		
			Roots: (M) 63 *μ*g/mL; (E) 63 *μ*g/mL		
			Stem: (M) 63 *μ*g/mL; (E) 63 *μ*g/mL		

*Aloe ferox* Mill.	Leaves	*E. coli*	(W) > 8000 *μ*g/mL	Not toxic in human epithelial kidney cells	[73]

*Aloe marlothii* A.Berger	Leaves	*E. coli*	(M) 1250 *μ*g/mL	Not determined	[75]
		*P. aeruginosa*	(M) 1250 *μ*g/mL		

*Apodytes dimidiata* E.Mey ex am.	Not stated	*E. coli*	(A) 2500 *μ*g/mL	Not determined	[76]
		*P. aeruginosa*	(A) 310 *μ*g/mL		

*Artemisia afra* Jacq. Ex Willd.	Leaves	*E. coli*	(W) 3000 *μ*g/mL	Not toxic in human epithelial kidney cells	[74]

*Ballota africana* (L.) Benth.	Leaves	*K. pneumoniae*	(M) 438 *μ*g/mL; (W) 379 *μ*g/mL	Non-toxic in *Artemia* lethality assay	[41]
		*P. mirabilis*	(M) 4278 *μ*g/mL		[40]

*Bolosanthus speciosis* (Bolus) Harms	Leaves	*E. coli*	(A) 80 *μ*g/mL	LC_50_ = 53 *μ*g/mL in Vero cells	[77]

*Brachylaena discolor*	Not stated	*E. coli*	(A) 630 *μ*g/mL	Not determined	[76]
		*P. aeruginosa*	(A) 310 *μ*g/mL		

*Calpurnia aurea* (aiton) Benth.	Leaves	*E. coli*	(A) 40 *μ*g/mL	LC*50* = 57 *μ*g/mL in Vero cells	[77]

*Carpobrotus edulis* (L.) N.E. Br.	Leaves	*P. mirabilis*	(M) 205 *μ*g/mL; (W) 561 *μ*g/mL	Non-toxic in *Artemia* lethality assay	[40]
		*Proteus vulgaris*	(M) 670 *μ*g/mL; (W) 1246 *μ*g/mL		

*Cissius quadrangularis* (Linn.)	Stems	*E. coli*	(M) 1259 *μ*g/mL	Not determined	[75]
		*P. aeruginosa*	(M) 2500 *μ*g/mL		

*Clausena anisata* (Willd.) Hook ex Benth.	Not stated	*E. coli*	(A) 310 *μ*g/mL	Not determined	[76]
		*P. aeruginosa*	(A) 310 *μ*g/mL		

*Clerodendron glabrum* E.Mey	Not stated	*E. coli*	(A) 310 *μ*g/mL	Not determined	[76]
		*P. aeruginosa*	(A) 630 *μ*g/mL		

*Combretum kraussii* Hochst.	Leaves	*E. coli*	Root and bark: (E) 1560 *μ*g/mL; (W) 3125 *μ*g/mL	Not determined	[72]

*Cremaspora triflora* (Thonn.) K.Schum.	Leaves	*E. coli*	(A) 50 *μ*g/mL	LC_50_ = 14 *μ*g/mL in Vero cells	[77]

*Cryptocarya latifolia* Sond.	Bark	*E. coli*	(M) 4000 *μ*g/mL; (W) > 8000 *μ*g/mL; (D) > 8000 *μ*g/mL; (EA) > 8000 *μ*g/mL; (H) > 8000 *μ*g/mL	Not determined	[78]
		*P. aeruginosa*	(M) 4000 *μ*g/mL; (W) > 8000 *μ*g/mL; (D) > 8000 *μ*g/mL; (EA) 2000 *μ*g/mL; (H) 4000 *μ*g/mL		

*Curtisia dentata* (Burm.f) C.A. Sm.	Not stated	*E. coli*	(A) 600 *μ*g/mL	Not determined	[79]
		*P. aeruginosa*	(A) 600 *μ*g/mL		

*Cussonia spicata* Thunb.	Bark	*E. coli*	(M) 1250 *μ*g/mL	Not determined	[75]
		*P. aeruginosa*	(M) 1250 *μ*g/mL		

*Cussonia zuluensis* Strey.	Not stated	*E. coli*	(A) 880 *μ*g/mL	Not determined	[79]
		*P. aeruginosa*	(A) 880 *μ*g/mL		

*Cyathea dregei* (Kunze.) R.M.Tyron	Not stated	*E. coli*	(A) 310 *μ*g/mL	Not determined	[76]
		*P. aeruginosa*	(A) 310 *μ*g/mL		

*Dicerocaryum ericarpum* (Decne.)	Shoots	*E. coli*	(M) 1250 *μ*g/mL	Not determined	[75]
		*P. aeruginosa*	(M) 1250 *μ*g/mL		

*Ekebergia capensi* Sparrm.	Leaves	*E. coli*	(M) 1000 *μ*g/mL; (W) > 8000 *μ*g/mL; (D) 1000 *μ*g/mL	Non-toxic in *Artemia* lethality assay	[80]

*Ekebergia pterophylla* (C.DC) Hofmeyr	Leaves	*E. coli*	(M) 1000 *μ*g/mL; (W) > 8000 *μ*g/mL; (D) 4000 *μ*g/mL	Non-toxic in *Artemia* lethality assay	[80]

*Elaeodendron croceum* (Thunb.) DC.	Leaves	*E. coli*	(A) 110 *μ*g/mL	LC_50_ = 5 *μ*g/mL in Vero cells	[77]

*Euclea crispa* (Thunb.) Gürke	Leaf	*E. coli*	(M) 1750 *μ*g/mL; (D) 1750 *μ*g/mL; (EA) 1280 *μ*g/mL	Not determined	[81]
		*P. aeruginosa*	(M) 2000 *μ*g/mL; (D) 2000 *μ*g/mL; (EA) 1500 *μ*g/mL		

*Euclea natalensis* A. DC.	Leaf	*E. coli*	(M) 1250 *μ*g/mL; (D) 1750 *μ*g/mL; (EA) 1380 *μ*g/mL	Not determined	[81]
		*P. aeruginosa*	(M) 1000 *μ*g/mL; (D) 2000 *μ*g/mL; (EA) 2000 *μ*g/mL		

*Eucomis autumnalis* (Mill.) Chitt.	Bulb	*E. coli*	(M) > 8000 *μ*g/mL; (W) > 8000 *μ*g/mL; (D) > 8000 *μ*g/mL; (EA) > 8000 *μ*g/mL; (H) > 8000 *μ*g/mL	Not determined	[78]
		*P. aeruginosa*	(M) > 8000 *μ*g/mL; (W) > 8000 *μ*g/mL; (D) > 8000 *μ*g/mL; (EA) > 8000 *μ*g/mL; (H) 2000 *μ*g/mL		

*Ficus sur* Forssk.	Root and bark	*E. coli*	Root and bark: (E) 1560 *μ*g/mL; (W) 4600 *μ*g/mL	Not determined	[72]

*Gymnosporia senegalensis* (Lam.) Loes.	Roots	*E. coli*	(M) 156 *μ*g/mL; (W) 312 *μ*g/mL; (E) 156 *μ*g/mL; (A) 312 *μ*g/mL	Not determined	[82]

*Hydnora africana* Thunb.	Bark	*E. coli*	(M) > 8000 *μ*g/mL; (W) > 8000 *μ*g/mL; (D) > 8000 *μ*g/mL; (EA) > 8000 *μ*g/mL; (H) > 8000 *μ*g/mL	Not determined	[78]
		*P. aeruginosa*	(M) 2000 *μ*g/mL; (W) > 8000 *μ*g/mL; (D) > 8000 *μ*g/mL; (EA) > 8000 *μ*g/mL; (H) > 8000 *μ*g/mL		

*Heteromorpha arborescens* (Spreng.) Cham & Schltdl.	Leaves	*E. coli*	(A) 180 *μ*g/mL	LC_50_ = 81 *μ*g/mL in Vero cells	[77]

*Hetromorpha trifoliata* Wendl. Eckl. & Zeyh.	Not stated	*E.coli*	(A) 630 *μ*g/mL	Not determined	[76]
		*P. aeruginosa*	(A) 630 *μ*g/mL		

*Heteropyxis natalensis* Harv.	Leaves	*E. coli*	(M) 382 *μ*g/mL	Non-toxic in *Artemia* lethality assay	[71]

*Hypericim roeperianum* G.W.Schimp. ex A. Rich.	Leaves	*E. coli*	(A) 130 *μ*g/mL	LC_50_ = 66 *μ*g/mL in Vero cells	[77]

*Hypoxis hemerocallidea* Fisch.Mey. & avé-Lall.	Leaves	*E. coli*	(M) 4000 *μ*g/mL; (W) 4000 *μ*g/mL; (D) > 8000 *μ*g/mL; (EA) > 8000 *μ*g/mL; (H) > 8000 *μ*g/mL	Not determined	[78]
		*P. aeruginosa*	(M) > 8000 *μ*g/mL; (W) > 8000 *μ*g/mL; (D) > 8000 *μ*g/mL; (EA) > 8000 *μ*g/mL; (H) > 8000 *μ*g/mL		

*Indigofera daleoides* Harv.	Whole plant	*E. coli*	(M) 78 *μ*g/mL; (E) 146 *μ*g/mL; (A) 78 *μ*g/mL	Not dertermined	[82]

*Indigofera frutescens* Linn. f.	Not stated	*E. coli*	(A) 160 *μ*g/mL	Not determined	[76]
		*P. aeruginosa*	(A) 310 *μ*g/mL		

*Jatropha zeheri* Sond.	Root	*E. coli*	(M) 630 *μ*g/mL	Not determined	[75]
		*P. aeruginosa*	(M) 2500 *μ*g/mL		

*Kigelia africana* (Lam.) Benth.	Leaves	*E. coli*	(M) 827 *μ*g/mL; (W) 681 *μ*g/mL	Non-toxic in *Artemia* lethality assay	[71]
		*K. pneumoniae*	(M) 965 *μ*g/mL; (W) 663 *μ*g/mL		[41]
		*P. mirabilis*	(M) 2483 *μ*g/mL; (W) 285 *μ*g/mL		[40]

*Leucosidea sericea* Eckl. & Zeyh.	Not stated	*E. coli*	(A) 80 *μ*g/mL	Not determined	[76]
		*P. aeruginosa*	(A) 20 *μ*g/mL		

*Lippia javanica* (Burm.f.) Spreng.	Leaves	*E. coli*	(M) 439 *μ*g/mL; (W) 192 *μ*g/mL	Non-toxic in *Artemia* lethality assay	[71]
		*K. pneumoniae*	(M) 538 *μ*g/mL; (W) 654 *μ*g/mL		[41]
		*P. mirabilis*	(M) 313 *μ*g/mL; (W) 1873 *μ*g/mL		[40]
		*Proteus vulgaris*	(M) 926 *μ*g/mL; (W) 1728 *μ*g/mL		[40]

*Maesa lanceolata* Forssk.	Leaves	*E. coli*	(A) 40–310 *μ*g/mL	LC_50_ = 2.4 *μ*g/mL in Vero cells	[76, 77]
		*P. aeruginosa*	(A) 20–310 *μ*g/mL		

*Melletia grandis* (E.Mey.) Skeels	Not stated	*E. coli*	(A) 310 *μ*g/mL	Not determined	[76]
		*P. aeruginosa*	(A) 310 *μ*g/mL		

*Melia azedarach* L.	Not stated	*E. coli*	(A) 310 *μ*g/mL	Not determined	[76]
		*P. aeruginosa*	(A) 630 *μ*g/mL		

*Morus mesozygia* Stapf.	Leaves	*E. coli*	(A) 70 *μ*g/mL	LC_50_ = 41 *μ*g/mL in Vero cells	[77]

*Nymania capensis* (Thunb.) Lindb.	Leaves	*E. coli*	(M) > 8000 *μ*g/mL; (W) > 8000 *μ*g/mL; (D) > 8000 *μ*g/mL	Non-toxic in *Artemia* lethality assay	[80]

*Ozoroa insignis* Delile	Stem bark	*E. coli*	(M) 156 *μ*g/mL; (W) 156 *μ*g/mL; (E) 156 *μ*g/mL; (A) 156 *μ*g/mL	Not determined	[82]

*Pelargonium sidoides* DC.	Leaves	*E. coli*	(W) > 8000 *μ*g/mL	Non-toxic in *Artemia* lethality assay	[73]

*Pittosporum viridiflorum* Sims	Leaves	*E. coli*	(A) 110 *μ*g/mL	LC_50_ = 55 *μ*g/mL in Vero cells	[74]

*Pelargonium fasiculata* (L.) Alton	Leaves	*K. pneumoniae*	(M) 374 *μ*g/mL; (W) 432 *μ*g/mL	Non-toxic in *Artemia* lethality assay	[41]
		*P. mirabilis*	(M) 806 *μ*g/mL; (W) 1843 *μ*g/mL		[40]

*Ptaerocarpus angolensis* DC.	Bark	*E. coli*	(M) 630 *μ*g/mL	Not determined	[75]
		*P. aeruginosa*	(M) 2500 *μ*g/mL		

*Ptaeroxylon obliquim (*Thunb.) Radlk.	Leaves	*K. pneumoniae*	(M) 1977 *μ*g/mL	Non-toxic in *Artemia* lethality assay	[41]
		*P. mirabilis*	(M) 239 *μ*g/mL; (W) 487 *μ*g/mL		[40]
		*Proteus vulgaris*	(M) 511 *μ*g/mL; (W) 727 *μ*g/mL		

*Prunus africana* (Hook. f.) Kalkman	Roots	*E. coli*	(M) > 8000 *μ*g/mL; (W) > 8000 *μ*g/mL; (D) > 8000 *μ*g/mL; (EA) > 8000 *μ*g/mL; (H) > 8000 *μ*g/mL	Not determined	[78]
		*P. aeruginosa*	(M) > 8000 *μ*g/mL; (W) > 8000 *μ*g/mL; (D) > 8000 *μ*g/mL; (EA) > 8000 *μ*g/mL; (H) > 8000 *μ*g/mL		

*Punica granatum* L.	Roots	*E. coli*	(M) 78 *μ*g/mL; (W) 78 *μ*g/mL; (E) 78 *μ*g/mL; (A) 78 *μ*g/mL	Not determined	[82]

*Rhicinus communis* Linn.	Leaves and stem	*E. coli*	(M) 400 *μ*g/mL	Not determined	[75]
		*P. aeruginosa*	(M) 780 *μ*g/mL		

*Rhoicissus rhomboidea* (E. Mey ex Harv.) Planch.	Leaves	*E. coli*	(M) 306 *μ*g/mL; (W) 333 *μ*g/mL	Non-toxic in *Artemia* lethality assay	[71]

*Rhoicissus tridentata* (L.f.) Wild & R.B.Drumm.	Roots	*E. coli*	(M) > 8000 *μ*g/mL; (W) > 8000 *μ*g/mL; (D) > 8000 *μ*g/mL; (EA) > 8000 *μ*g/mL; (H) > 8000 *μ*g/mL	Not determined	[78]
		*P. aeruginosa*	(M) 2000 *μ*g/mL; (W) > 8000 *μ*g/mL; (D) > 8000 *μ*g/mL; (EA) > 8000 *μ*g/mL; (H) > 8000 *μ*g/mL		

*Riccinus communis* L.	Leaves	*E. coli*	(A) 13130 *μ*g/mL	Not determined	[83]
		*P. aeruginosa*	(M) 14670 *μ*g/mL; (E) 16670 *μ*g/mL		
		*K. pneumoniae*	(M) 12670 *μ*g/mL; (A) 11670 *μ*g/mL		

*Sacrostemma viminale* R. Br.	Stem	*E. coli*	(M) 1250 *μ*g/mL	Not determined	[75]
		*P. aeruginosa*	(M) 1250 *μ*g/mL		

*Schkuhria pinnata* (Lam.)	Shoots	*E. coli*	(M) 310 *μ*g/mL	Not determined	[75]
		*P. aeruginosa*	(M) 1250 *μ*g/mL		

*Schotia bractopetalia* Sond.	Leaves	*E. coli*	(M) 505 *μ*g/mL; (W) 312 *μ*g/mL; (E) 491 *μ*g/mL; (A) 312 *μ*g/mL	Non-toxic in *Artemia* lethality assay	[71]

*Spirostachys africana* Sond.	Stem bark	*E. coli*	(M) 156 *μ*g/mL; (W) 312 *μ*g/mL; (E) 156 *μ*g/mL; (A) 156 *μ*g/mL	Not determined	[82]

*Syzygium cordatum* (Hochst.)	Leaves and bark	*E. coli*	(M) 499 *μ*g/mL; (W) 790 *μ*g/mL	Non-toxic in *Artemia* lethality assay	[71]
		*K. pneumoniae*	(M) 312 and 387 *μ*g/mL (bark and leaves respectively); (W) 387 and 335 *μ*g/mL (bark and leaves respectively)		[41]
		*P. mirabilis*	(M) 969 and 474 *μ*g/mL (bark and leaves respectively); (W) 932 and 49 *μ*g/mL (bark and leaves respectively)		[40]
		*P. vulgaris*	(M) 751 and 641 *μ*g/mL (bark and leaves respectively); (W) 1325 and 658 *μ*g/mL (bark and leaves respectively)		[40]

*Strychnos madagascariensis* Poir.	Leaves	*E. coli*	(M) 580 *μ*g/mL; (W) 593 *μ*g/mL	Non-toxic in *Artemia* lethality assay	[71]

*Strychnos mitis* S.Moore	Not stated	*E. coli*	(A) 40 *μ*g/mL	Not determined	[76]
		*P. aeruginosa*	(A) 160 *μ*g/mL		

*Sutherlandia frutescens* (L.) R.Br.	Leaves	*E. coli*	(W) > 8000 *μ*g/mL	Not toxic in human epithelial kidney cells	[73]

*Terminalia phanerophlebia* Engl. & Diels,	Not stated	*E. coli*	(A) 80 *μ*g/mL	Not determined	[79]
		*P. aeruginosa*	(A) 80 *μ*g/mL		

*Terminalia pruinoides* M.A. Lawson	Leaves	*E. coli*	(M) 278 *μ*g/mL; (W) 624 *μ*g/mL	Non-toxic in *Artemia* lethality assay	[71]
		*K. pneumoniae*	(M) 432 *μ*g/mL; (W) 531 *μ*g/mL		[41]
		*P. mirabilis*	(M) 313 *μ*g/mL; (W) 224 *μ*g/mL		[40]
		*P. vulgaris*	(M) 926 *μ*g/mL; (W) 379 *μ*g/mL		

*Terminalia sambesiacia* Engl. & Diels.	Not stated	*E. coli*	(A) 60 *μ*g/mL	Not determined	[79]
		*P. aeruginosa*	(A) 60 *μ*g/mL		

*Terminalia sericea* Burch. ex DC.	Leaves	*E. coli*	(M) 396 *μ*g/mL; (W) 276 *μ*g/mL	Non-toxic in *Artemia* lethality assay	[71]
		*K. pneumoniae*	(M) 254 *μ*g/mL; (W) 318 *μ*g/mL		[41]
		*P. mirabilis*	(M) 417 *μ*g/mL; (W) 103 *μ*g/mL		[40]
		*P. vulgaris*	(M) 508 *μ*g/mL; (W) 520 *μ*g/mL		

*Trichilia dregeana* Sond.	Leaves	*E. coli*	(M) 1000 *μ*g/mL; (W) > 8000 *μ*g/mL; (D) 8000 *μ*g/mL	Non-toxic in *Artemia* lethality assay	[80]
		*E. faecalis*	(M) 1500 *μ*g/mL; (W) > 8000 *μ*g/mL; (D) 4000 *μ*g/mL		

*Trichilia emetica* Vahl.	Leaves	*E. coli*	(M) 1000 *μ*g/mL; (W) > 8000 *μ*g/mL; (D) 8000 *μ*g/mL	Non-toxic in *Artemia* lethality assay	[80, 84]

*Tulbaghia violaceae* Harv.	Leaves	*E. coli*	Roots: (M) 387 *μ*g/mL; Leaves: (M) 30 *μ*g/mL	LC_50_ = 772 *μ*g/mL in *Artemia* lethality assay	[71]
		*K. pneumoniae*	Roots; (M) 526 *μ*g/mL; (W) 613 *μ*g/mL		[41]
		*P. mirabilis*	Leaves: (W) 125 *μ*g/mL		[40]

*Turraea floribunda* Hochst.	Leaves	*E. coli*	(M) 4000 *μ*g/mL; (W) > 8000 *μ*g/mL; (D) 4000 *μ*g/mL	Non-toxic in *Artemia* lethality assay	[80]

*Turraea obtusifolia* Hochst.	Leaves	*E. coli*	(M) 2000 *μ*g/mL; (W) > 8000 *μ*g/mL; (D) 8000 *μ*g/mL	Non-toxic in *Artemia* lethality assay	[80]

*Vepris reflexa* I. Verd.	Not stated	*E. coli*	(A) 600 *μ*g/mL	Not determined	[79]
		*P. aeruginosa*	(A) 1250 *μ*g/mL		

*Warburgia salutaris* (Bertol.f.) Chiov.	Leaves and bark	*E. coli*	Leaves: (M) 239 *μ*g/mL; (W) 304 *μ*g/mL	Non-toxic in *Artemia* lethality assay	[71]
		*K. pneumoniae*	(M) 624 *μ*g/mL (bark); (W) 677 *μ*g/mL (bark)		[41]
		*P. mirabilis*	(M) 623 and 465 *μ*g/mL (bark and leaves respectively); (W) 417 *μ*g/mL (bark)		[40]
		*P. vulgaris*	(M) 450 and 688 *μ*g/mL (bark and leaves respectively); (W) 1203 *μ*g/mL (leaves)		[40]

*Ximenia caffra* Sond.	Stem bark	*E. coli*	(M) 156 *μ*g/mL; (W) 312 *μ*g/mL; (E) 312 *μ*g/mL; (A) 312 *μ*g/mL	Not determined	[82]

*Zanthoxylem capensis* (Thunb.) Harv.	Not stated	*E. coli*	(A) 310 *μ*g/mL	Not determined	[76]
		*P. aeruginosa*	(A) 310 *μ*g/mL		

*Ziziphus murconata* Willd.	Bark	*E. coli*	(M) 2500 *μ*g/mL	Not determined	[75]
		*P. aeruginosa*	(M) 1250 *μ*g/mL		

## Data Availability

All data are included within this study and are also available from the corresponding author on request.
